# Determinants of life satisfaction and self-rated health in Iranian children and adolescents: a structure equation model

**DOI:** 10.1186/s12887-021-03044-w

**Published:** 2022-01-03

**Authors:** Pooneh Angoorani, Zohreh Mahmoodi, Hanieh-Sadat Ejtahed, Ramin Heshmat, Mohammad Esmaeil Motlagh, Mostafa Qorbani, Roya Kelishadi

**Affiliations:** 1 Chronic Diseases Research Center, Endocrinology and Metabolism Population Sciences Institute, Tehran University of Medical Sciences, Tehran, Iran; 2 Social Determinants of Health Research Center, Alborz University of Medical Sciences, Karaj, Iran; 3 Obesity and Eating Habits Research Center, Endocrinology and Metabolism Clinical Sciences Institute, Tehran University of Medical Sciences, Tehran, Iran; 4 Department of Pediatrics, Ahvaz Jundishapur University of Medical Sciences, Ahvaz, Iran; 5 Non-communicable Diseases Research Center, Alborz University of Medical Sciences, Karaj, Iran; 6 Endocrinology and Metabolism Research Center, Endocrinology and Metabolism Clinical Sciences Institute, Tehran University of Medical Sciences, Tehran, Iran; 7 Department of Pediatrics, Child Growth and Development Research Center, Research Institute for Primordial Prevention of Non-communicable Disease, Isfahan University of Medical Sciences, Isfahan, Iran

**Keywords:** Life satisfaction, Self-rated health, CASPIAN, Children, Adolescents, Youth

## Abstract

**Background:**

Life satisfaction (LS) and self-rated health (SRH) are related with health outcomes. It is expected that these items are also related to healthy behaviors. Therefore, this study was conducted in order to find out the main determinants of LS and SRH in nationwide representative sample of Iranian children and adolescents.

**Methods:**

This study was performed on 13,834 students aged 7–18 years who were selected by multistage, stratified cluster sampling method from 30 provinces of Iran. Life satisfaction and SRH were assessed through a questionnaire based on World Health Organization-Global School-based Student Health Survey protocols. Path analysis was applied to evaluate the relationships among the study variables using the structural modeling.

**Results:**

Life satisfaction was directly affected by age (− 0.037 in boys & -0.028 in girls); sedentary time (0.055 in boys & 0.048 in girls); school satisfaction (0.249 in boys & 0.250 in girls); and well-being (0.186 in boys & 0.176 in girls). Self-rated health was directly affected by LS (0.28 in boys & girls) and school satisfaction (0.21 in boys & 0.22 in girls); and indirectly affected by age (− 0.046 in boys & -0.017 in girls); sedentary time (− 1.99 in boys & -0.145 in girls); family size (− 0.005 in boys & -0.014 in girls); and socio-economic status (0.015 in boys & 0.058 in girls).

**Conclusions:**

This study indicated that school satisfaction had the greatest positive direct effect on both LS and SRH.

**Supplementary Information:**

The online version contains supplementary material available at 10.1186/s12887-021-03044-w.

## Introduction

Childhood and adolescence are considered to be a life phase in which future health patterns for adulthood are being initiated. It is characterized as a period of relatively good physical and mental health, high life satisfaction, and low mortality. During this developmental outlook, low life satisfaction and bad health condition can have a diverse effect on the improvement of developmental challenges related to adolescence and ultimately lead to several long-term negative consequences in adulthood [1, 2]. Infectious disease, malnutrition, and mortality have been lowered in children and adolescent populations, however, shifting attention to chronic diseases, mental health problems, obesity, and physical illness, which their prominence during childhood and adolescence is very concerning [3]. Life satisfaction (LS) and self-rated health (SRH), assess different dimensions of individuals’ own health that show some non-biomedical factors of general well-being framed by individual, familial and social dimensions [4]. Life satisfaction can be defined as the degree to which an individual judges the overall quality of his life-as-a-whole favorably [5]. Self-rated health is considered a powerful global indicator of health and mortality that show the effects of some non-biomedical factors, such as lifestyle, psychosocial, and socio-demographic conditions [6]. An increasing body of literature has been conducted regarding the relationship between LS and factors related to social conditions such as the individual’s living conditions or society-level, economic well-being, and physical and mental health on adult populations [7–9]. However, there is limited practical investigation on SRH and LS for children or adolescents. Therefore, the present study aimed to comprehend main determinants of LS and SRH in a nationwide representative sample of Iranian children and adolescents according to the path analysis, as a powerful statistical model in evaluating a complex cluster of dependent variables [10].

## Methods

### Procedures and participants

This nationwide study was conducted in urban and rural areas of Iran in 2015 as the fifth national survey of a school-based surveillance program entitled the Childhood and Adolescence Surveillance and Prevention of Adult Non-communicable disease (CASPIAN-V) study. Data was checked at the district level by academic supervisors (expert of school health) and controlled by national supervisors and operators. Detailed description of the sampling and data collection methods are published previously [11].

The study population consisted of students aged 7–18 years in primary and secondary schools in urban and rural areas across the country. Totally, 13,834 students were selected by multistage, stratified cluster sampling method from 30 provinces of the country (48 clusters of 10 students in each province). Stratification was performed in each province according to the residence area (urban/rural) and level of education (primary/ secondary). The sampling size was proportional to population in each urban or rural area with equal sex ratio. Cluster sampling with equal clusters was used in each province to reach the necessary sample size. Clusters were determined at the level of schools, including 10 statistical units (students and their parents) in each cluster.

The protocols of the present study were assessed and approved by the Research and Ethics Council of Isfahan University of Medical Sciences (Project Number: 194049). Written informed consent and verbal consent was obtained from the parents and students, respectively [11]. This study was conducted according to the guidelines laid down in the Declaration of Helsinki, and all procedures involving human subjects were approved by Ethics Committee of Isfahan University of Medical Sciences.

### Anthropometric measurements

Trained healthcare staff conducted the anthropometric measurement according to the standard protocol [12]. Height was measured without shoes to the nearest 0.5 cm using stadiometer (Seca, Hamburg, Germany). Weight was measured with light clothes to the nearest 0.1 kg by scale (Seca, Hamburg, Germany). Body mass index (BMI) was calculated as weight (kg) divided by square of height (m^2^). Waist circumference (WC) was measured at the point midway between the lower border of the rib cage and the iliac crest to the nearest 0.1 cm [11]. Waist to height ration was calculated by dividing WC to height.

### Questionnaires

Two sets of the questionnaires were used for students and their parents. The questionnaires were obtained from Global School Student Health Survey (GSHS) that translated to Persian [13]. The reliability and validity of the Persian version of questionnaires was approved in the previous studies [14, 15].

The student questionnaire included questions regarding body image, psychosocial environment of school, dietary habits, life-style habits, and violence behavior. Trained personnel completed questionnaires in a calm atmosphere inside the schools; the whole process was supervised and controlled by a team of health care professionals.

**Fig. 1:**
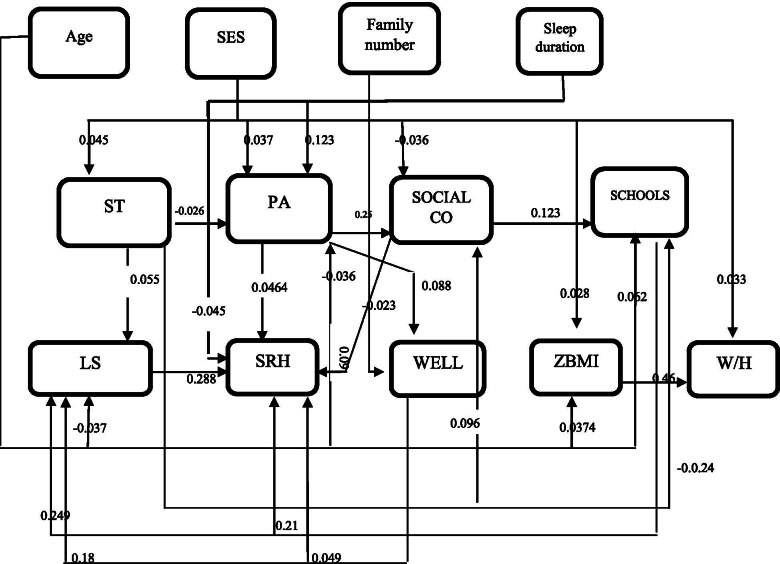
Path analysis diagram of association of general characteristics with life satisfaction and self-rated health for boys

**Fig. 2:**
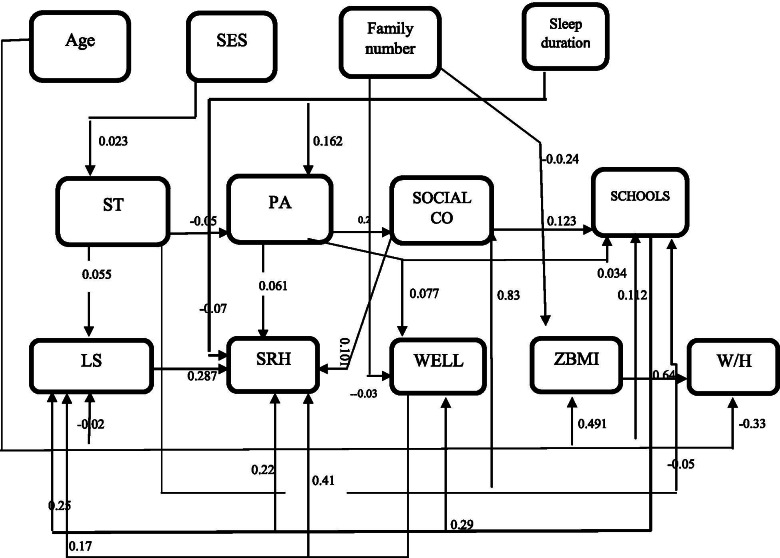
Path analysis diagram of association of general characteristics with life satisfaction and self-rated health for girls

Issues such as family composition, economic and socio-demographic factors, genetic determinants (family history of hypertension, diabetes, and obesity), post-birth data (birth weight, breastfeeding, and type of complementary food), and family dietary habits were included in the parent’s questionnaire which was completed by the parents themselves (Figs. 1 and 2).

### Measuring tools

The questionnaire of the World Health Organization-Global School-based Student Health Survey (WHO-GSHS) was used to assess aggressive behaviors, LS, SRH and counseling with family members. Demographic information on age, gender, residence area, family-based characteristics, living with parents, parental level of education, possessing a family private car and type of home gathered through interview with students. We prepared Persian versions of standardized questionnaires, which were designed based on world health organization models. The validity and reliability of the questionnaires were confirmed through the previous study [16].

**Life satisfaction (LS)** was assessed through a single item. Students were asked to indicate their degree of life satisfaction by using a tenth-point scale from 1 = very dissatisfied to 10 = very satisfied. Fewer than 6 responses were aligned to dissatisfaction and responses of equal and upper 6 were defined as satisfaction.

**Self-rated health (SRH)** was assessed through a single item, “how would you describe your general state of health?”; the categories of response were “perfect”, “good”, “bad,” and “very bad”. For statistical analysis, “perfect and good” responses were considered as “good SRH”. Moreover, the preference of participant in consulting with father, mother, and sister/brother and friends were asked for further analyses.

**Physical activity** was assessed through a validated questionnaire including weekly frequency of leisure time physical activity outside the school during the past week, and having sufficient physical activity was defined as at least 30 min of exercise per day that led to sweating and large increases in breathing or heart rate [13, 17].

**Sedentary time** Sedentary behavior is considered as the waking time in a sitting, reclining, or lying posture on screen time (television, video game playing, computer using) or reading, characterized by an energy expenditure less than 1.5 metabolic equivalents [18]. The sedentary time was accessed using a validated questionnaire [13, 17].

**Socioeconomic status (SES)** was calculated through a validated questionnaire [16] included questions about the following socioeconomic indicators: (a) parental level of education (illiterate: score 1, less than high school: score 2, high school graduate: score 3, academic education: score 4); (b) parental occupational status (unemployed: score 1, worker/farmer: score 2, governmental employee: score 3, self-employed: score 4); (c) number of inhabitants in home, and (d) possessing a family private car (yes/no). It should be noted that for questions (a) and (b) (i.e. parental occupational status and level of education) data from the parent or legal guardian with a higher occupational status/education was considered.

**School satisfaction** was assessed through a validated questionnaire regarding the overall satisfaction with school life experience including interest in learning tasks, attitude to homework, school environment, relationships with teachers and classmates [16].

**Social contact** was assessed through a validated questionnaire regarding social relationship, the number of friends and time spend with them [16].

**Well-being** was considered as the overall satisfaction with relationships with family members and friends and current life conditions**.**

### Statistical analysis

All variables were checked for normality and expressed as means (standard deviation, SD). Student’s two-tailed *t* test was used to compare the mean differences of characteristics between boys and girls. Pearson correlation was applied to examine the relationships between the study variables and to implement the subsequent structural modeling. Path analysis was applied to examine the causal framework. Path analysis includes causal modeling, analysis of covariance structures, and latent variable models. We conceptualized this model according to literature review and our previous studies using causal association in the form of a directed acyclic graph between the explanatory variables with outcomes (LS-SRH). This model is a generalization of multivariate multiple regression that allows one to estimate the strength and sign of direction and indirection association for complicated causal schemes with multiple dependent and independent variables [19, 20]. Path standardized coefficients (β) as the effect sizes of associations were calculated. Goodness of fit (GOF) indices (e.g. The Root Mean Square Error of Approximation (RMSEA), the goodness of fit index (GFI), the adjusted GFI) were applied for assessing of fitness of the model [21]. All of the statistical analysis was performed using IBM, AMOS and STATA 11.0 (STATA Corp, College Station, TX). *P*-value less than 0.05 was considered as statistically significant.

## Results

Totally, 13,834 students (50.7% boys and 49.3% girls; mean age: 12.2 ± 3.15 years) were assessed in this study. There were no significant differences in age, SES, anthropometric indices, ST, school satisfaction, sleep duration, wellbeing, SRH and LS between boys and girls. However, physical activity and social contact were significantly higher in boys than in girls (*P* < 0.001). Table 1 shows the correlation among variables according to sex. In both sexes, SRH was positively correlated with age (r = 0.029 for boys and r = 0.63 for girls), school satisfaction (r = 0.312 for boys and r = 0.326 for girls), and social contact (r = 0.034 for boys and r = 0.044 for girls), but negatively correlated with LS (r = − 0.367 for boys and r = − 0.370 for girls), physical activity (r = − 0.057 for boys and r = − 0.070 for girls), sedentary time (r = − 0.034 for boys and r = − 0.035 for girls), and sleep duration (r = − 0.097 for boys and r = − 0.123 for girls). In both sexes, LS was positively correlated with SES, sleep duration, social contact, and wellbeing, but negatively correlated with age, ZBMI and school satisfaction (the amounts of “r” are illustrated in the Table 2). In both genders the most correlation was observed between SRH and LS (r = − 0.367 for boys and r = − 0.370 for girls). The direct effect of all variables according to sex is presented in Table 2. In both genders, age showed a positive direct effect on school satisfaction and ZBMI; physical activity had positive direct effect on well-being, social contact and SRH; sedentary time showed positive direct effect on LS and social contact but negative direct effect on physical activity and school satisfaction; school satisfaction and well-being had positive direct effects on both LS and SRH; social contacts had positive direct effect on SRH; life satisfaction had positive direct effect on SRH. In boys, SES had positive direct effect on physical activity, ZBMI, sedentary time and waist to high ratio and negative effect on social contact while social contacts had positive direct effect on school satisfaction. These findings were insignificant in girls.

**Table 1:** Matrix for Pearson correlation among characteristics

		SRH	LS	Age	W/H	SES	PA	ST	SLEEP DU	ZBMI	SC	Well being	SCHOOL S
SRH	Boy	–	−.37^**^	.03^*^	−.001	.003	−.06^**^	−.03^**^	−.01^**^	.02	.03^**^	−.19^**^	.31^**^
	Girl	–	−.37^**^	.63^**^	−.001	.001	−.07^**^	−.03^**^	−.12^**^	.03^**^	.04^**^	.18^**^	.33^**^
LS	Boy		–	−.63^**^	.009	.03^**^	.04^**^	.067^**^	.07^**^	−.02	.025^*^	.25^**^	−.31^**^
	Girl		–	−.06^**^	.04^**^	.03^**^	.04^**^	.067^**^	.07^**^	−.006	.27^*^	.26^**^	−.31^**^
Age	Boy			–	−.022	−.011	−.04^**^	.49^**^	−.06^**^	.37^**^	.003	−.38^**^	.06^**^
	Girl			–	−.017	−.03^**^	−.03^*^	.05^**^	−.07^**^	.49^**^	−.009	−.05^**^	.11^**^
W/Ht	Boy				–	.05^**^	−.02	.25^*^	−.005	.46^**^	−.008	.009	−.02
	Girl				–	.35^**^	−.01	−.002	−.006	.48^**^	−.007	−.01	−.007
SES	Boy					–	−.04^**^	−.04^**^	−.019	.04^**^	−.04^**^	.01	.01
	Girl					–	−.03^*^	.02	−.015	.018	−.015	.02	−.001
PA	Boy						–	−.02^*^	.125^**^	−.013	.25^**^	.10^**^	−.05^**^
	Girl						–	−.05^**^	.16^**^	−.002	.20^**^	.09^**^	−.05^**^
ST	Boy							–	.26^*^	.26^*^	.09^**^	.025^*^	−.03^**^
	Girl							–	.03^*^	.05^**^	.07^**^	.03^**^	−.06^**^
SLEEP DU	Boy								–	−.03^*^	.18^**^	.07^**^	−.19^**^
	Girl								–	−.03^*^	.18^**^	.05^**^	−.18^**^
ZBMI	Boy									–	.02	−.02	.03^**^
	Girl									–	−.014	−.02	.06^**^
SC	Boy										–	.05^**^	−.13^**^
	Girl										–	.04^**^	−.11^**^
Well being	Boy											–	−.30^**^
	Girl											–	−.29^**^
SCHOOL S	Boy												–
	Girl												–

**. Correlation is significant at the 0.01 level (2-tailed).*. Correlation is significant at the 0.05 level (2-tailed)

This analysis has been conducted on 7009 boys and 6825 girls

LS = Life satisfaction, SLEEPDU=Sleep duration, SES=Socio economic statues,

ST = Sedentary time, PA = Physical activity, SCHOOLS=School satisfaction, SC=Social contact, SRH = self-rated health, W/Ht = Waist to high, ZBMI = Z score of Body Mass Index

**Table 2:** Direct effects of variables in Iranian children and adolescents

	Boy			Girl		
	βestimate	standardized estimate	T-VALUE	βestimate	standardized estimate	T-VALUE
Age→ PA	−0.011	−0.036	3.39*	−0.02	−0.045	1.03
Age→ LS	−0.026	− 0.037	3.34*	−0.020	− 0.028	2.48*
Age → SCHOOLS	0.495	0.062	5.25*	0.089	0.112	9.38*
Age→W/Ht	− 0.004	−0.001	0.65	−0.006	− 0.334	29.14*
Age →ZBMI	0.125	0.374	33.72*	0.146	0.491	46.62*
SES → PA	0.037	0.037	3.15*	0.123	0.324	1.34
SES → ZBMI	0.030	0.028	2.20*	0.021	0.012	0.765
SES → SC	−0.133	− 0.036	3.15*	−0.003	−0.002	1.67
SES → ST	0.031	0.045	3.78*	0.016	0.023	1.97
SES → W/Ht	0.002	0.033	3.08*	0.010	0.022	1.43
FS → WELLBEING	−0.07	− 0.023	1.99*	−0.107	− 0.033	2.82*
FS → ZBMI	− 0.052	−0.065	1.09	−0.032	− 0.061	4.79*
SLEEPDU→PA	0.098	0.123	10.36*	0.126	0.162	13.55*
SLEEPDU→SRH	−0.028	− 0.045	4.12*	−0.043	− 0.07	6.35*
PA → SRH	0.036	0.046	4.09*	−0.0403	0.061	4.49*
PA → WELLBEING	0.007	0.088	4.24*	0.383	0.077	6.64*
PA → SC	0.91	0.250	21.65*	0.717	0.206	17.44*
PA → SCHOOLS	− 0.049	−0.019	1.59	0.089	0.034	2.763*
ST → LS	0.181	0.055	4.96*	0.166	0.048	4.26*
ST → PA	−0.036	− 0.026	2.18*	−0.078	− 0.054	4.49*
ST → SC	0.497	0.096	8.30*	0.417	0.083	6.97*
ST → SCHOOLS	−0.087	− 0.024	2.02*	−0.221	− 0.058	4.81*
SC → SRH	0.019	0.091	8.12*	0.023	0.101	9.09*
SC → SCHOOLS	0.086	0.123	10.27*	0.009	0.201	1.45
SCHOOLS→LS	0.224	0.249	21.32*	0.223	0.250	20.89*
SCHOOLS→SRH	0.064	0.210	17.91*	0.067	0.223	18.83*
SCHOOLS→WELL BEING	0.171	0.203	1.34	0.547	0.29	25.02*
WELLBEING→LS	0.089	0.186	16.00*	0.082	0.176	14.74*
WELLBEING→SRH	0.007	0.049	4.24*	0.006	0.41	3.55*
LS → SRH	0.097	0.288	24.93*	0.096	0.287	24.81*
ZBMI→W/Ht	0.029	0.46	43.5*	0.043	0.648	56.49*

*= SIGNIFICANT LS = Life satisfaction, SLEEPDU=Sleep duration, SES=Socio economic status, ST = Sedentary time, PA = Physical activity, FS = Family Size, SCHOOLS=School satisfaction, SC=Social contact, SRH = self-rated health, W/Ht = Waist to high, ZBMI = Z score of Body Mass Index

The direct, indirect and total effect of variables on LS and SRH is illustrated in Table 3. LS was directly affected by age (− 0.037 in boys & -0.028 in girls), sedentary time (0.055 in boys & 0.048 in girls), school satisfaction (0.249 in boys & 0.250 in girls), and well-being (0.186 in boys & 0.176 in girls). The other variables including sleep duration, physical activity, family size, SES, and social contact had slight indirect effect on LS. Among studied variables, only age and sedentary time had both direct and indirect effect on LS. In both genders, school satisfaction had the greatest direct effect and social contact had the greatest indirect effect on LS.

**Table 3:** Total effects of variables on life satisfaction and self-rated health in Iranian children and adolescents

	Life Satisfaction						Self-rated health					
	Boy			Girl			Boy			Girl		
	Direct effect	Indirect effect	Total effect	Direct effect	Indirect effect	Total effect	Direct effect	Indirect effect	Total effect	Direct effect	Indirect effect	Total effect
Age	− 0.037*	0.0146*	− 0.022*	− 0.028*	0.033*	− 0.005*	–	− 0.0046*	− 0.0046*	–	− 0.017*	−.017*
ST	0.055*	− 0.0029*	0.052*	0.048*	− 0.011*	0.037*	–	− 1.99*	− 1.99*	–	− 0.145*	− 0.145*
School satisfaction	0.249*	–	0.249*	0.250*	0.049*	.299*	0.210*	0.0717*	0.28*	0.223*	0.21*	0.433*
Well being	0.186*	–	0.186*	0.176*	–	0.176*	0.049*	0.052*	.101*	0. 41*	.048*	.458*
Sleep duration	–	0.0029*	0.0029*	–	0.005*	0.005*	− 0.045*	.003*	− 0.042*	− 0.07*	0.09*	.02*
PA		0.0236*	0.0236*	–	0.03*	0.03*	0.046*	0.016*	0.062*	0.061*	.077*	.138*
Family size	–	− 0.0004*	− 0.0004*	–	− 0.005*	− 0.005*	–	− 0.005*	− 0.005*	–	− 0.0137*	−0.0137*
SES	–	0.002*	0.002*	–	0.001*	0.001*	–	0.015*	0.015*	–	0.058*	0.058*
Social contact	–	0.03*	0.03*	–	0.636*	0.636*	0.091*	0.0338*	0.124*	0.101*	0.053*	0.154*

*= SIGNIFICANT ST = Sedentary time, PA = Physical activity, SES=Socio economic status

In case of SRH, school satisfaction, well-being, sleep duration, and physical activity had both direct and indirect effect on SRH in both genders. Self-rated health was directly affected by LS (0.288 in boys & 0.287 in girls); and indirectly affected by age (− 0.046 in boys & -0.017 in girls), sedentary time (− 1.99 in boys & -0.145 in girls), family size (− 0.005 in boys & -0.014 in girls), and SES (0.015 in boys & 0.058 in girls). In both genders, LS had the greatest direct effect on SRH. After that, school satisfaction had the greatest direct effect on SRH in boys and well-being had the greatest direct effect on SRH in girls (Supplementary Figures).

The results of model fitness with accepted range for evaluating the validity of the model are characterized in Table 4. There are a variety of fit indices to evaluate the model. All of them demonstrated that the model had acceptable fitting.

**Table 4:** The results of model fitness

MODEL	X^2^	df	CFI	GFI	NFI	RMSEA	IFI
Boy	668.93	49	0.95	0.97	0.94	0.04	0.95
Girl	408.29	45	0.964	0.97	0.960	0.03	0.964

## Discussion

This study was designed to evaluate the important determinants of LS and SRH in nationwide representative sample of Iranian children and adolescents examining the direct and indirect effects of different physical and psychosocial status of children on their LS and SRH.

In this investigation, LS and SRH showed a strong correlation with each other. Life satisfaction is an assessment of overall well-being and SRH indicates the children and adolescents’ perception of their health and applies as an index for their physiological conditions [3, 22]. The relationship of LS and SRH was reported in previous researches [23–25]. Studies showed that individuals with a healthy lifestyle have better SRH and higher LS [26]. The evaluation of LS and SRH from a holistic approach is very complex and so many factors influence them. In this study, we tried to determine the most effective factors in LS and SRH in children and adolescents. Through the path analytic method we found that among studied variables, school satisfaction and well-being had the greatest positive direct effect on both LS and SRH. Moreover, our results showed that the association between SES and LS was attributable to indirect effects through physical activity, ZBMI, social contact and sedentary time in boys and through sedentary time in girls. It means that higher SES is associated with more physical activity, BMI, social contact and sedentary time in children and adolescents which in turn could lead to more LS. On the other hand, family size showed slight indirect effect on LS through its negative effect on wellbeing and BMI. Physical activity and social contact were significantly higher in boys than in girls in our study. Previous studies also suggested that adolescent and adult males are more active than females in leisure-time, although not all were consistent [27, 28]. An explanation for this finding relies on the lack of appealing public places for physical activity practice for females in some countries. Moreover, cultural and religious beliefs can also have a significant impact on these differences. In consistence with our findings, previous studies have shown that satisfaction at school has positive effect on students’ satisfaction with life by decreasing both the school drop-out rate and disruptive behaviors [29, 30]. School satisfaction was shown to reduce the students’ illness, raising positive emotions, life satisfaction and academic success [31]. On average, subjects who have higher social contact, take part in physical activities, spend more time with family and friends seem to have greater satisfaction with life [5]. As expected, all factors which decrease psychosocial stress in adolescents including having good relationships with family and friends, positive school environment and higher SES can improve subjective well-being and LS [32–35]. The literature indicated that LS was predicted by variables related to population health and there was a strong correlation between LS and health behaviors [36–38]. It is suggested that the individuals with normal BMI reported more LS than obese person [39]. The study conducted by Ford et al. regarding the association of BMI with health-related quality of life showed that after adjusting for confounders, thin and obese people, had lower SRH and LS than people with normal BMI [39]. Furthermore, an inverse association was shown between LS with negative health-related behaviors such as physical inactivity [36]. These results are in line with our findings and show that appropriate BMI and physical activity as two main health behaviors seem to have positive effect on LS.

This study provides new evidence about the determinants of SRH and LS as important indicators for students’ health care in a large sample of Iranian children and adolescents. However, the cross-sectional design of this study had a limited ability for demonstrating a causal relationship and understanding the possible changes of subscales over time and their predictive validity. Moreover, self-report measure of some potentially important factors including personality variables, increases the risk of biases in this method. The variables related to mental health and satisfaction were also obtained through questionnaires, so these measurements may contain some errors and probability of misclassification is existed. Future studies with longitudinal design and several data collections are suggested in order to verify the main determinants of life satisfaction in children and adolescents to improve health, both in adolescence and later in life.

## Conclusion

The findings of the current study indicated that LS and SRH of children and adolescents are directly or indirectly affected by many factors including age, physical activity, socio economic status, sleep duration, well-being, family size, social contacts, sedentary time and school satisfaction. Among these variables school satisfaction had the greatest positive direct effect on both LS and SRH. Therefore, it needs to notice the significance of these factors on the life and health satisfaction of children and adolescents and health-promoting programs should be designed according to the observed associations to improve their health outcomes.

## Supplementary Information

**Additional file 1.**

## Data Availability

The datasets used and/or analyzed during the current study are available from the corresponding author on reasonable request.

## Abbreviations

BMI: body mass index
LS: Life satisfaction
PA: Physical activity
SES: Socioeconomic status
SRH: self-rated health
ST: Sedentary time
