# Supplementary material for: Determinants of life satisfaction and self-rated health in Iranian children and adolescents: a structure equation model
Source: BMC Pediatr. 2022 Jan 3;22:4. doi: 10.1186/s12887-021-03044-w (PMC8721968; doi:10.1186/s12887-021-03044-w)
Supplement: Supplementary file 1 — Additional file 1. [file 12887_2021_3044_MOESM1_ESM.docx]

Family number

Age

Sleep duration

SES

0.123

-0.036

0.037

0.045

ST

SCHOOLS

SOCIAL CO

PA

-0.026

0.123

0.25

-0.036

0.055

0.033

-0.045

0.088

0.028

-0.023

0.062

0.0464

0.09

W/H

WELL

SRH

LS

ZBMI

0.288

0.46

ZBMI

0.0374

0.096

-0.037

ZBMI

-0.0.24

0.21

0.249

0.18

0.049

Figure1- Path analysis diagram of association of general characteristics with life satisfaction and self-rated health for boys.

Sleep duration

0.162

0.023

-0.0.24

0.123

0.2

ST

0.034

0.077

0.055

0.83

-0.07

0.287

0.64

0.25

0.101

-0.05

0.17

0.061

0.112

-0.02

W/H

ZBMI

WELL

SRH

LS

SCHOOLS

SOCIAL CO

PA

Family number

SES

Age

--0.03

0.491

-0.33

0.22

0.41

-0.05

0.29

Figure 2- Path analysis diagram of association of general characteristics with life satisfaction and self-rated health for girls.
